# Alcohol use disorders and the heart

**DOI:** 10.1111/add.14703

**Published:** 2019-07-15

**Authors:** Ed Day, James H. F. Rudd

**Affiliations:** ^1^ Institute for Mental Health, School of Psychology, University of Birmingham, and Honorary Consultant in Addiction Psychiatry, Solihull Integrated Addiction Service UK; ^2^ Division of Cardiovascular Medicine University of Cambridge, Honorary Consultant Cardiologist, Addenbrooke's Hospital Cambridge UK

**Keywords:** Alcohol, arrhythmia, cardiac, cardiomyopathy, cardiovascular, hypertension, mortality

## Abstract

Alcohol use is an important preventable and modifiable cause of non‐communicable disease, and has complex effects on the cardiovascular system that vary with dose. Observational and prospective studies have consistently shown a lower risk of cardiovascular and all‐cause mortality in people with low levels of alcohol consumption when compared to abstainers (the ‘J’‐shaped curve). Maximum potential benefit occurs at 0.5 to one standard drinks (7–14 g pure ethanol) per day for women (18% lower all‐cause mortality, 95% confidence interval (CI) = 13–22%) and one to two standard drinks (14–28 g ethanol) per day for men (17% lower all‐cause mortality, 95% CI = 15–19%). However, this evidence is contested, and overall the detrimental effects of alcohol far outweigh the beneficial effects, with the risk of premature mortality increasing steadily after an average consumption of 10 g ethanol/day. Blood pressure (BP) is increased by regular alcohol consumption in a dose‐dependent manner, with a relative risk for hypertension (systolic BP > 140 mm Hg or diastolic > 90 mm Hg) of 1.7 for 50 g ethanol/day and 2.5 at 100 g/day. Important reductions in BP readings can be expected after as little as 1 month of abstinence from alcohol. Heavy alcohol consumption in a binge pattern is associated with the development of acute cardiac arrhythmia, even in people with normal heart function. Atrial fibrillation is the most common arrhythmia associated with chronic high‐volume alcohol intake, and above 14 g alcohol/day the relative risk increases 10% for every extra standard drink (14 g ethanol). Ethanol and its metabolites have toxic effects on cardiac myocytes, and alcoholic cardiomyopathy (ACM) accounts for a third of all cases of non‐ischaemic dilated cardiomyopathy. Screening people drinking alcohol above low‐volume levels and delivering a brief intervention may prevent the development of cardiovascular complications. Although people with established cardiovascular disease show improved outcomes with a reduction to low‐volume alcohol consumption, there is no safe amount of alcohol to drink and patients with ACM should aim for abstinence in order to optimize medical treatment.

## Introduction

The harmful effects of alcohol on the heart began to appear in the medical literature in the 19th century. In 1886 the director of the Munich Institute of Pathology, Otto Bollinger, described an alcohol intake of up to 12 litres of beer per day in a series of 42 cases of cardiac hypertrophy, where many patients were employed by breweries or in liquor stores 1. In his 1901 textbook of pathology he described what came to be known as ‘Plethoric Munich Beer Heart’:

‘idiopathic cardiac hypertrophy usually with dilatation… most frequently found in certain forms of chronic alcoholism (beer drinkers), whereby plethora and toxic influences become effective as pathogenic factors’.

Alcohol use is one of the four most common preventable and modifiable causes of major non‐communicable diseases 2, and the globalization of production and marketing of alcohol have increased both the amount of world‐wide consumption and the harms associated with it 3. The Alcohol Group of the Global Burden of Disease (GBD) study found that alcohol was the seventh leading risk factor for both deaths and disability‐adjusted life years (DALYs), accounting for 2.2% [95% uncertainty interval (UI) 1.5–3.0] of age‐standardized female deaths and 6.8% (5.8–8.0) of age‐standardized male deaths 4. In 2016 alcohol use led to 2.8 million deaths world‐wide and was the leading risk factor for premature death and disability among people aged 15–49 years, with nearly 9% of all attributable DALYs for men and more than 2% for women. By evaluating all associated relative risks for alcohol use, the GBD team found that drinking no alcohol minimized the overall risk to health 4. However, although it is now well established that excessive alcohol consumption has an adverse effect on health and mortality 5, epidemiological studies have also suggested some potential benefits of low levels of consumption, including reductions in coronary heart disease events, ischaemic cerebrovascular events and cardiovascular and total mortality 6. These beneficial effects are contested, and need to be set against by the negative effects of alcohol contributing to other diseases, accidents and injury and on society as a whole 7.

This review summarizes the epidemiological evidence for the impact of alcohol on the heart at low, medium and high levels, before focusing on clinical presentations of people who consume alcohol at higher levels or in a binge pattern. There is a particular focus on hypertension, cardiac arrhythmias and alcoholic cardiomyopathy. Consideration is given to the clinical presentation, pathological mechanisms, assessment, management and prognosis of each disorder, as well as the evidence of approaches to managing alcohol use disorders in this population.

## Epidemiology

There are difficulties in interpreting data relating to the health consequences of alcohol consumption. There is a lack of world‐wide consensus as to the amount of alcohol in a standard alcoholic drink. In the United States a ‘standard drink’ contains 14 g of pure alcohol, but a ‘unit’ of alcohol in the United Kingdom is classified as 8 g of pure alcohol, and a standard drink in other countries may contain as much as 20 g of alcohol 8. This review will adopt the US definition of a standard drink unless stated otherwise. Definitions of ‘low’, ‘moderate’ and ‘hazardous’ alcohol consumption have also varied, but most investigators have now settled on the following broad definition: low‐risk drinking = two or fewer drinks/day for men and one drink/day or less for women, moderate‐risk drinking = three to four drinks/day for men and two to three drinks/day for women, high‐risk drinking = five or more drinks/day for men and four or more drinks/day for women and binge drinking = five or more drinks/occasion for men and four or more drinks/occasion for women.

Other factors complicating epidemiological studies of alcohol consumption include lack of adequate control for confounding factors such as cigarette smoking, general health and socio‐economic status; problems with the accurate assessment of alcohol consumption (e.g. assessment over fewer than 30 days used to assess life‐time risk of morbidity and mortality and a failure to assess both quantity and frequency of consumption); counting both former drinkers and occasional drinkers as abstainers (which may make moderate drinkers seem healthier if ill health is the reason for abstention or occasional drinking); and combining occasional drinkers with moderate drinkers 9. In fact, Rehm has argued that methodological issues render the utility of cohort studies assessing the relationship between alcohol use and all‐cause mortality as ‘almost meaningless’ 10.

## Alcohol and Cardiovascular Mortality: The J‐Shaped Curve

**Figure 1:**
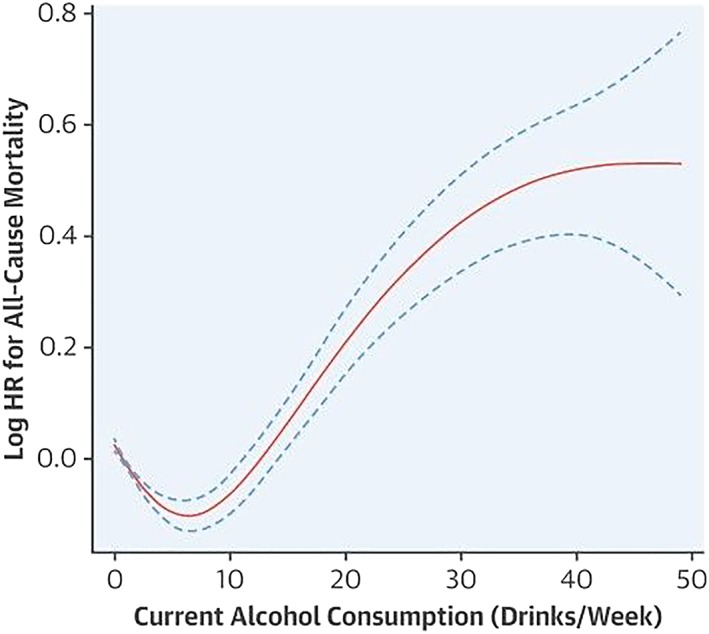
Dose–response association of current alcohol consumption with all‐cause mortality 5. This figure is used with permission of Elsevier, licence number 4591410186372. [Colour figure can be viewed at wileyonlinelibrary.com]

**Figure 2:**
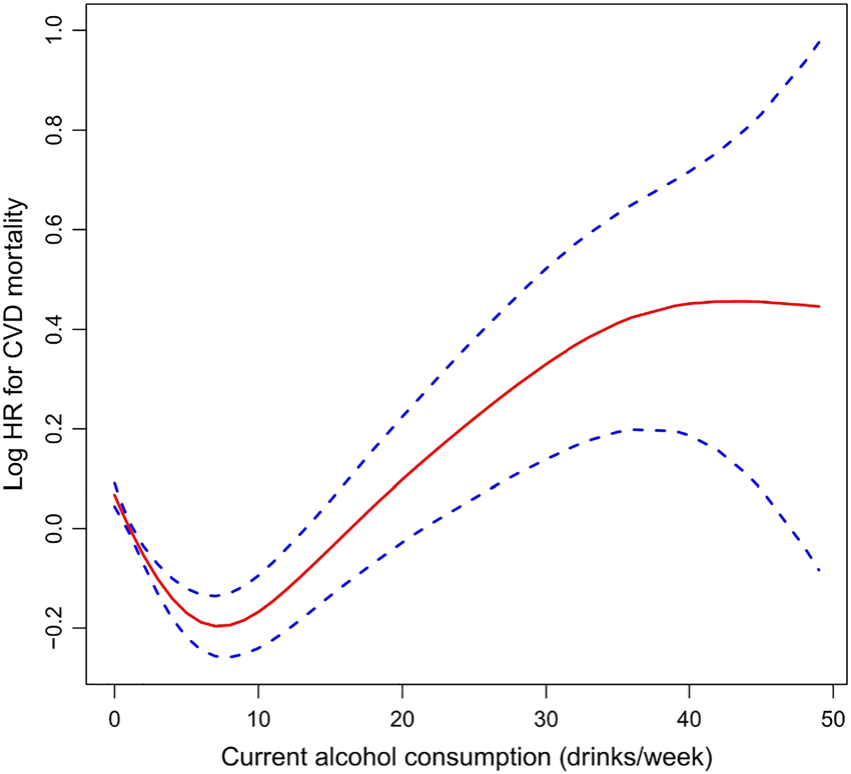
Dose–response association of current alcohol consumption with cardiovascular disease‐specific (CVD) mortality 5. This figure is used with the permission of Elsevier, licence number 4591410186372. [Colour figure can be viewed at wileyonlinelibrary.com]

Observational and prospective studies have consistently shown a lower risk of all‐cause and cardiovascular mortality in people with low levels of alcohol consumption when compared with abstainers, with the highest risks occurring in people with high levels of consumption (the ‘J‐shaped’ or ‘U‐shaped’ curve) 5, 6, 11, 12. Xi and colleagues 5 examined the association between alcohol consumption and mortality risk in US adults, using data from the National Health Interview Surveys of 333 247 participants ≥ 18 years of age and categorizing participants according to self‐reported alcohol consumption patterns. Compared with life‐time abstainers, individuals who were classified as light or moderate consumers were at reduced risk of all‐cause mortality and cardiovascular mortality, but that risk increased significantly with heavy alcohol consumption. This is seen in the J‐shaped curves in Figs 1 and 2. A meta‐analysis of 34 studies including more than a million people found a maximum potential benefit at half to one drink daily for women [18% lower all‐cause mortality; 95% confidence interval (CI) = 13–22%] 13. In men, maximal potential was seen at one to two drinks/day, with an associated reduction of 17% (95% CI = 15–19%) in all‐cause mortality 13. Consumption of more than 2.5 drinks/day in women and four drinks/day in men was associated with higher death rates in a dose‐dependent manner.

Beverages such as red wine that are high in polyphenols have anti‐oxidant, anti‐inflammatory and antiplatelet effects. However, ethanol appears to be the main driver of potential health benefits at low doses and the toxic effects at high doses. Increased high‐density lipoprotein (HDL), reduced plasma viscosity, decreased fibrinogen concentration, increased fibrinolysis, decreased platelet aggregation and coagulation and enhanced endothelial function are some of the potentially beneficial mechanisms 11, 14.

One criticism of this literature is that low‐volume drinkers may appear healthier only because the ‘abstainers’ with whom they are compared are not drinking because of pre‐existing medical conditions (so‐called ‘sick quitters’) or disease caused by prior high levels of alcohol consumption. A meta‐analysis of 87 studies investigating alcohol use and mortality risk replicated the classic J‐shaped curve, with low‐volume drinkers (1.3–24.9 g ethanol/day) having reduced mortality risk [relative risk (RR) = 0.86, 95% CI = 0.83, 0.90] 15. Occasional drinkers (< 1.3 g/day) had similar mortality risk (RR = 0.84, 95% CI = 0.79, 0.89) and former drinkers had elevated risk (RR = 1.22, 95% CI = 1.14, 1.31). However, after adjustment for abstainer biases and quality‐related study characteristics, no significant reduction in mortality risk was observed for low‐volume drinkers (RR = 0.97, 95% CI = 0.88, 1.07). Restriction of the analysis to higher‐quality, bias‐free studies also failed to show a reduced mortality risk for low‐volume alcohol drinkers. Risk estimates for occasional drinkers were similar to those for low‐ and medium‐volume drinkers 15.

Any cardioprotective effect of regular low‐volume alcohol consumption disappears as soon as episodes of excessive drinking occur. One meta‐analysis found that individuals with irregular heavy drinking episodes of ≥ 60 g pure alcohol per occasion (> 12×/year but < five×/week) had a 45% increased morbidity and mortality risk from ischaemic heart disease compared with individuals with a regular, moderate alcohol use pattern, even after adjusting for study type, subject age and smoking, and exclusion of former drinkers to control for sick quitters 16. Overall, the detrimental effects of alcohol far outweigh the beneficial effects, and the risk of premature mortality increases steadily after 10 g average daily consumption in European populations 17.

## Clinical Presentations Relating to the Heart

When considering the heart and cardiovascular system, high doses of alcohol can have both acute (depression of cardiac contractility, cardiac rhythm disturbances, arterial hypertension, sudden death) and chronic effects (ventricular dysfunction, atrial dysfunction, arrhythmia, alcoholic cardiomyopathy and heart failure) 11. In addition, chronic high doses of alcohol are associated with the development of hypertension, coronary and peripheral atherosclerosis, changes in lipid profile and an increased risk of all forms of stroke. Heavy use of alcohol is often accompanied by the use of other harmful drugs such as tobacco and cocaine, with synergistic deleterious effects 18, 19.

## Hypertension

Hypertension is most commonly defined as systolic blood pressure (BP) > 140 mmHg or diastolic BP > 90 mmHg. The 2015 GBD study put hypertension as the leading single risk factor for morbidity and mortality, responsible for 10.7 million deaths and 211.8 million DALYs world‐wide 20. Cross‐sectional studies, cohort studies and meta‐analytical reviews all point to BP being increased by regular alcohol consumption in a dose‐dependent manner 6, 21. For example, a multinational meta‐analysis showed a linear relationship between alcohol and BP, with a relative risk for hypertension of 1.7 for 50 g ethanol/day and 2.5 at 100 g/day 21. However, gender is an important modifier of the alcohol threshold level for harm. The Physician's Health Study found an apparent protective effect for women between two and four drinks/week and one drink/day, but the relationship in men was linear even at low levels of average consumption 22. This gender‐specific result does not have an obvious biological explanation, but one possibility is the finding that more binge drinking episodes occur among men at lower levels of average consumption, and these are particularly related to increases in hypertension incidence and risk 23.

Assessment and treatment of alcohol‐associated hypertension follows standard clinical guidelines 24, with ambulatory BP monitoring being used to confirm the diagnosis. A significant reduction of alcohol use is essential, alongside medications such as ACE inhibitors, calcium channel blockers and diuretics. Important improvements in BP readings can be expected after as little as 1 month of abstinence from alcohol, with one study demonstrating a 7.2 mmHg reduction in mean BP in previously heavy drinkers 25.

The link between alcohol consumption and hypertension makes it a key part of the World Health Organization (WHO) goals to reduce non‐communicable disease mortality. In a meta‐analysis of 36 trials, a decrease in alcohol intake reduced BP in people who drank more than two drinks/day, but not in those consuming two or fewer drinks/day 26. The American Society of Hypertension and the International Society of Hypertension recommend that men limit their alcohol consumption to no more than two drinks a day and women to no more than one drink a day 27. To put the importance of BP control into perspective at a population level, a 2‐mmHg increase in BP increases mortality from stroke by 10% and from coronary artery disease (CAD) by 7% 28.

## Arrhythmia

Heavy alcohol consumption in a binge pattern is associated with the development of acute cardiac arrhythmia, even in those with normal heart function. The term ‘holiday heart’ was coined by Ettinger in 1978 to describe ‘an acute cardiac rhythm and or conduction disturbance associated with heavy ethanol consumption in a person without other clinical evidence of heart disease and disappearing without evident residual, with abstinence’ 29. The authors noted a seasonal rise in the incidence of arrhythmias during a period of traditional binge drinking (Christmas and New Year). Atrial fibrillation (AF) was the most common arrhythmia noted, but atrial flutter, junctional tachycardia, isolated premature ventricular complexes, isolated premature atrial complexes, paroxysmal atrial tachycardia and ventricular tachycardia were also described.

Alcohol may act together with coexistent electrolyte disturbance and/or acute illnesses such as infections to produce supraventricular arrhythmias 30. Sudden death in alcohol misuse (SUDAM) is increasingly recognized, and case–series have described heavy drinkers who die suddenly with no autopsy findings other than fatty liver change 31. There are several possible (but unproven) causes of such deaths, and a ventricular arrhythmia unmasked by persistent heavy alcohol use is one possibility. When compared with deaths due to sudden arrhythmic death syndrome, the SUDAM group are significantly older and have a 10‐fold greater incidence of fatty liver change 32. Heavy alcohol consumption is associated with QT interval prolongation 33, and alcohol dependence commonly presents alongside polysubstance use and psychiatric morbidity where QT‐prolonging medications may be part of the clinical picture, e.g. tricyclic antidepressants, selective serotonin reuptake inhibitors (SSRIs), lithium and methadone 34. The risk of ventricular tachycardia and sudden cardiac death is lower in individuals with modest alcohol intake (two to six drinks/week) than those with high intake (21–35 drinks/week) and binge drinkers. This may be attributable to the protective effects of low‐to‐moderate alcohol consumption against coronary artery disease as described above 13. Arrhythmias may also be manifestations of an underlying alcoholic cardiomyopathy, and so a holistic cardiovascular assessment in important.

Daily alcohol intake is associated with a shorter right atrial effective refractory period (AERP) and increased atrial flutter in patients less than 60 years old 35. The shorter right AERP may allow the right atrium to sustain a rapid rate in response to an initiating rhythm or allow propagation of an appropriately timed premature atrial complex 35. Atrial fibrillation is the most common arrhythmia associated with chronic high‐volume alcohol intake. Above a safe threshold of approximately one drink/day the relative risk of AF increases 10% for each drink per day 36. However, it appears that the relationship between chronic alcohol consumption and arrhythmias may be non‐linear, involving complex interactions between dose, drinking pattern, age and sex 34. For example, Psaty found alcohol was inversely related with incidence of AF in older adults with a lower level of drinking (two to three drinks/week) and a low incidence of binge drinking 37. Acute alcohol withdrawal in chronic alcohol users can increase cardiac sympathetic activity and reduce both heart rate variability and baroreflex sensitivity, promoting cardiac arrhythmias 34.

Assessment and treatment of arrhythmia in patients who are heavy users of alcohol can be challenging if abstinence, or at least a significant reduction in intake, is not achieved 38. Specifically, for atrial fibrillation, anticoagulation to reduce risk of stroke and ventricular rate control are central to the management strategy. In some cases, direct current cardioversion or electrophysiologically guided ablation techniques may be used, but ablation is known to be less successful in those who continue to drink alcohol, with higher AF relapse rates 39.

## Alcoholic Cardiomyopathy

Chronic excessive alcohol consumption is a leading cause of secondary dilated cardiomyopathy. However, partial or total recovery of cardiac function can occur if the disease is diagnosed early and further alcohol intake is reduced or halted. Alcoholic cardiomyopathy (ACM) accounts for 33% of all cases of non‐ischaemic dilated cardiomyopathy 40, and the prevalence is similar in males and females (alcohol consumption is higher in men, but women are more susceptible to its effects) 41.

**Figure 3:**
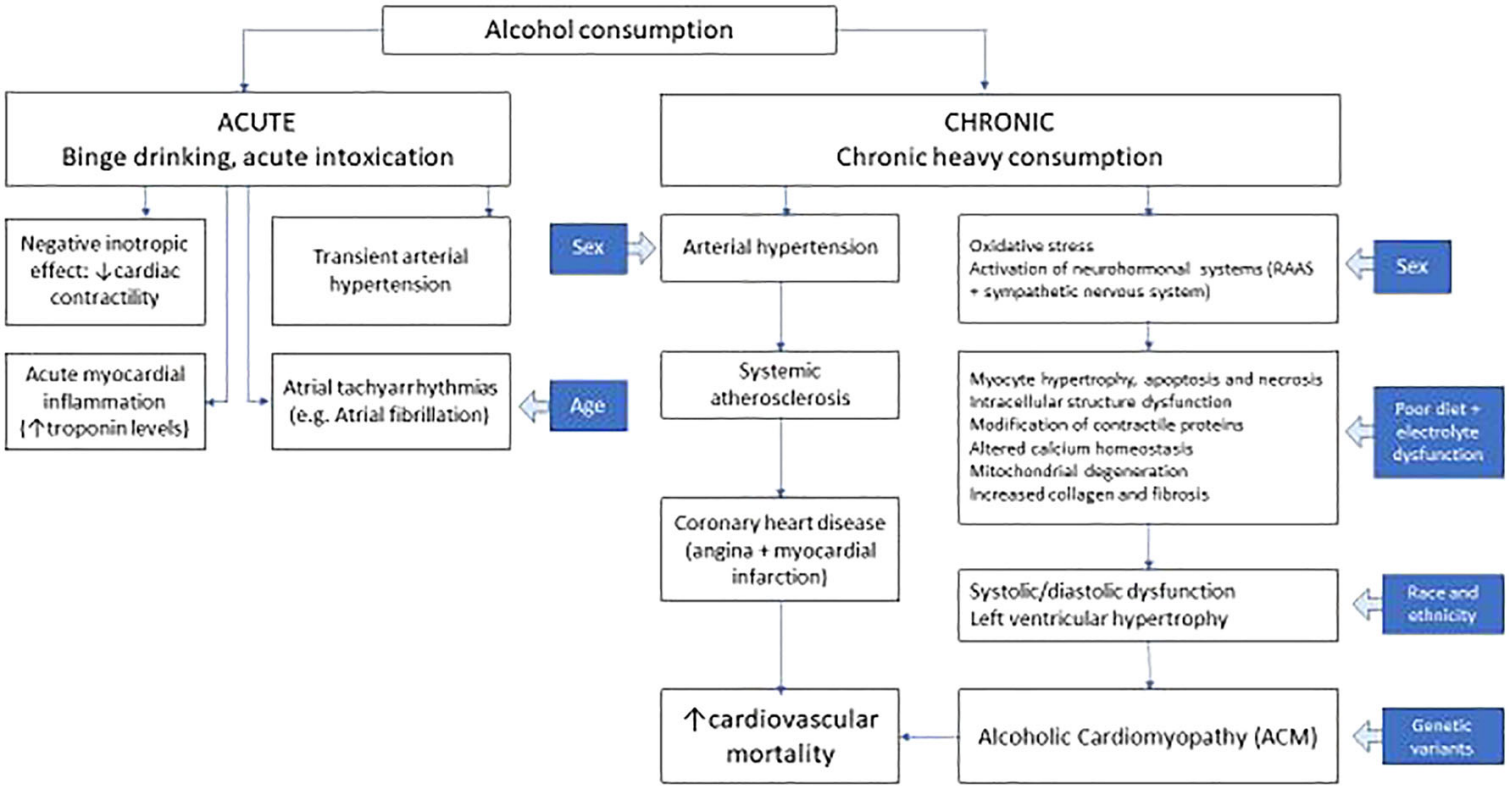
A summary of the effects of acute and chronic alcohol consumption on the heart and cardiovascular system. The blue boxes and arrows represent influences on susceptibility to alcohol‐related harm. Adapted from Piano 2017 14 and Mirijello *et al*. 2017 41. [Colour figure can be viewed at wileyonlinelibrary.com]

Alcohol can lead to excess free‐radical generation and oxidative stress through at least three mechanisms: ethanol metabolism to acetaldehyde and ethyl esters; effects on anti‐oxidant proteins and enzymes; and activation or alteration of neurohormonal systems such as the sympathetic nervous system or the renin–angiotensin–aldosterone system (RAAS) 14. Cardiac myocytes are excitable cells with complex signalling structures, and as such are highly sensitive to oxidative stress. Experimental models of chronic heart damage have many limitations, but over time heavy alcohol use directly impairs myocardial function by interfering with calcium homeostasis, mitochondrial function, and the structure and function of contractile proteins 41. Myocyte apoptosis and necrosis occurs, and although there is a low rate of regeneration after cell death, repair mechanisms involving hypertrophy of remaining cells can initially offset the effect of myocyte death 11. However, reduced levels of myofibrillar proteins, combined with the expression of different isoforms of myosin, result in depressed contraction 41. These changes eventually translate into increased left ventricular dilatation and mass, thinning and left ventricular dysfunction 34. Genetic variants in certain proteins/enzymes, variability in nutrition, ethnic and sex differences may also influence the occurrence of ACM 14, 41, 42 (see Fig. 3).

One‐third of men with a total life‐time dose of ethanol of more than 5 kg/kg have echocardiographic evidence of impaired diastolic filling, an effect that persists after correction for multiple confounders. This is proposed as the earliest sign of subclinical ACM 11, 43. Long‐term high alcohol consumption is also associated with systolic dysfunction in a dose‐dependent way. Prospective studies have shown that chronic alcohol consumption of more than 100 g/day in men and 80 g/day in women over a period of 10 years is associated with dose‐dependent effects on left ventricular function 44.

Regular, heavy alcohol use can lead to ACM, characterized by dilation and impaired contraction of one or both ventricles in the presence of normal or reduced ventricular wall thickness, with no other identified cause of cardiomyopathy 45. ACM is defined as an acquired dilated cardiomyopathy associated with long‐term heavy alcohol consumption (> 80 g per day over a period of at least 5 years). Typical histological findings are myofibrillar necrosis and fibrosis, with reduced myofibrils and giant mitochondria, findings which do not differentiate ACM from other forms of dilated cardiomyopathy.

Presenting symptoms relate to the reduction in cardiac output and are the same as chronic cardiac failure of any aetiology, i.e. shortness of breath on exertion, bilateral pitting oedema, fatigue, mental confusion, oliguria and nocturia. Physical examination may reveal a raised jugular venous pressure, third and/or fourth heart sound and a systolic murmur, and possibly a tachyarrhythmia such as AF. Diagnosis requires a long history of significant alcohol use and exclusion of other causes of dilated cardiomyopathy. Blood tests such as gamma‐glutamyl transferase (GGT), mean corpuscular volume (MCV), carbohydrate‐deficient transferrin (CDT) and ethyl‐gluconide may help to diagnose alcohol use disorder, coexisting liver disease and monitor abstinence. N‐terminal pro‐B‐type natriuretic peptide (NT pro‐BNP) levels are increasingly used to make a diagnosis of heart failure in patients with breathlessness, and may be helpful. A chest X‐ray may show cardiomegaly, pulmonary congestion and pleural effusions, depending on disease severity. An electrocardiogram may reveal coexisting atrial fibrillation, along with non‐specific ST and T‐wave changes. There are no tell‐tale electrocardiogram (ECG) features of ACM that can differentiate it from other causes of cardiomyopathy. Echocardiography is mandatory for diagnosis and to exclude other causes of heart failure. Typically, it shows biventricular dilatation and systolic and diastolic dysfunction. Occasionally, cardiac magnetic resonance imaging (MRI) is needed to confirm the diagnosis if echocardiographic images are suboptimal. Exclusion of contributing coronary artery disease with coronary angiography is often required.

The prevailing wisdom regarding treatment of ACM is total abstinence from alcohol to allow potential recovery of cardiac function. Unfortunately, no blood or imaging biomarkers of potential reversibility have been identified. Abstinence has been associated with improved LV function in small observational studies in some patients (e.g. 46). However, Nicolas *et al*. found significant cardiac improvement in both those achieving abstinence and those reducing consumption to less than 60 g ethanol/day, but cardiac function deteriorated in those who did not change drinking 47. As there is no safe amount of alcohol to drink, it is best to aim for abstinence in the first instance.

After abstinence, treatment of ACM should include beta‐blockers, ACE inhibitors, angiotensin receptor antagonists and diuretics, following standard guidelines 48. Patients with persistent severe ventricular dysfunction despite optimized pharmacological therapy should be evaluated for implantable cardioverter defibrillator (ICD) insertion and heart transplant if considered appropriate. Eating a balanced diet is important, and any nutritional deficiencies should be corrected. Vitamin supplementation with vitamin B12, vitamin B6 and folate may be needed, particularly for those with sustained heavy alcohol use. In the largest published follow‐up study of patients with ACM (*n* = 94, median follow‐up 59 months), approximately one‐third had a poor prognosis, whereas two‐thirds remained clinically stable. Atrial fibrillation, QRS width > 120 msec, and the absence of beta‐blocker therapy were associated with a worse outlook 49.

## Management of Alcohol Use Disorder and Cardiac Disorders

Interventions for alcohol use disorders exist along a spectrum 50. Screening people drinking at ‘at‐risk’ level and delivering a brief intervention may prevent the development of cardiovascular complications such as hypertension and arrhythmias. However, despite a strong evidence base for such interventions 51, they are rarely implemented in routine medical practice 52. Excessive consumption of alcohol is one of a number of modifiable causes of health problems in the developed world. As medical care moves towards a chronic disease model there have been calls to incorporate effective behaviour change strategies into medical practice, and a number have been identified for changing alcohol use 53.

Motivational Interviewing (MI) is a counselling style that aims to address the problem of ambivalence about change, and is both person‐centred and directional in its focus on goals such as changes in health behaviours 54. In a systematic review and meta‐analysis of the efficacy of MI throughout medical settings, 63% of the main outcome comparisons yielded statistically significant advantages favouring MI 55. MI produced a statistically significant and positive impact on amount of alcohol consumed, but this did not include any studies of populations with cardiac disease. Unlike other areas of medicine (e.g. surgery 56 or liver failure 57), there is little published literature on interventions tailored to reducing alcohol use in populations with cardiovascular disease.

Psychosocial interventions are efficacious in treating alcohol use disorder 58, and are the mainstay of treatment. The predominant treatment goal for alcohol dependence has traditionally been abstinence. Acamprosate and naltrexone have been shown to reduce the risk of returning to drinking when prescribed in conjunction with psychosocial interventions 59, and neither have cardiac‐related contraindications. Disulfiram works in a different manner (inhibition of acetaldehyde dehydrogenase enzyme leading to a build‐up of acetaldehyde and associated unpleasant physical effects), and its deterrent effect can improve the outcome of treatment when a third party is involved to assist with compliance 60. At the usual prescribed dose of 200–250 mg/day, the disulfiram‐alcohol interaction varies from mild flushing to distressing nausea, headache, dizziness and chest tightness. Fatal cases reported in the medical literature suggest that cardiac causes of death are common 61. In the early days of disulfiram use, patients were given a test dose of alcohol, and most patients developed some ECG changes during the reaction (e.g. QT‐prolongation). Hypotension and hypertension are also common, and the potentially fatal outcome of a disulfiram‐alcohol reaction in a patient with heart disease means that disulfiram is not usually offered to this group.

Abstinence from alcohol is likely to produce the best outcomes for established hypertension, arrhythmia and cardiomyopathy, but may not be a feasible or acceptable goal for many patients. Intervention strategies have now broadened to include reduction of alcohol intake, and even alcohol‐dependent people with loss of control over the past 12 months can reduce their drinking 62. Treatment programmes aiming at harm reduction help to establish self‐determined controlled drinking and use this as a bridge to long‐term abstinence 63, 64. Interventions aiming at reducing total alcohol consumption and/or modifying the drinking pattern are associated with positive effects in harmful, hazardous or alcohol‐dependent drinkers 65.

## Prognosis

### Secondary prevention

People with established cardiovascular disease show improved outcomes with a reduction to low‐volume drinking. A meta‐analysis of 16 351 patients with a previous diagnosis of CVD found a J‐shaped curve with maximal risk at two drinks/day 66. A similar relationship exists between alcohol and cardiovascular mortality in patients who have experienced a myocardial infarction 67, and low levels of alcohol are also associated with less atherosclerotic progression in coronary artery bypass grafts post‐coronary artery bypass grafting (CABG) 68.

### Mortality rates

The mortality risk may decline threefold if a person with a usual alcohol intake of 96 g ethanol/day cuts down to 36 g ethanol/day compared with a constant daily intake of 60 g ethanol/day 69. In one long‐term prospective cohort study (*n* = 850), 15% heavy drinking patients (defined as 50 g ethanol or more per drinking occasion or daily drinking) died after 10 years and 39.1% after 20 years, compared to low‐volume drinkers consuming less than 50 g ethanol per drinking occasion less than once per month (5.3% at 10 years and 19.7% at 20 years) 70. In contrast, significantly more relapsed patients (73%) died prematurely at the mean age of 48 years (mainly from heart attack or heart failure) compared to those without relapses (30%) in the 16‐year observation period.

### Conclusions

Although low‐volume alcohol consumption (one drink/day or less for women and two drinks for men) may confer some benefit in terms of reduced risk of ischaemic heart disease 71, alcohol use at higher levels is associated with increased cardiovascular risk and premature mortality. Occasional binge drinking (more than five drinks within a few hours, with intoxication as intended consequence) reverses any protection associated with low‐volume alcohol consumption. The main cardiovascular effects of long‐term alcohol consumption are hypertension, cardiac arrhythmia, cardiomyopathy and heart failure. Treatment for alcohol use disorder is best focused on abstinence from alcohol, although gains are often achieved by significant reduction in alcohol consumption or changes in pattern of drinking.

### Declaration of interests

None.
